# Clinical and financial outcomes of transplant recipients following emergency general surgery operations^☆^

**DOI:** 10.1016/j.sopen.2023.04.002

**Published:** 2023-04-15

**Authors:** Zachary Tran, Jonathan Lee, Shannon Richardson, Syed Shahyan Bakhtiyar, Lauren Shields, Peyman Benharash

**Affiliations:** a Cardiovascular Outcomes Research Laboratories, Division of Cardiac Surgery, Department of Surgery, University of California, Los Angeles, CA, United States of America; b Department of Surgery, Loma Linda University Health, Loma Linda, CA, United States of America

**Keywords:** Transplant, Emergency general surgery, Nationwide readmissions database, Entropy balancing

## Abstract

**Introduction:**

Due to immunosuppression and underlying comorbidities, transplant recipients represent a vulnerable population following emergency general surgery (EGS) operations. The present study sought to evaluate clinical and financial outcomes of transplant patients undergoing EGS.

**Methods:**

The 2010–2020 Nationwide Readmissions Database was queried for adults (≥18 years) with non-elective EGS. Operations included bowel resection, perforated ulcer repair, cholecystectomy, appendectomy and lysis of adhesions. Patients were classified by transplant history (*Non-transplant*, *Kidney/Pancreas*, *Liver*, *Heart/Lung*). The primary outcome was in-hospital mortality while perioperative complications, resource utilization and readmissions were secondarily considered. Multivariable regression models evaluated the association of transplant status on outcomes. Entropy balancing was employed to obtain a weighted comparison to adjust for intergroup differences.

**Results:**

Of 7,914,815 patients undergoing EGS, 25,278 (0.32 %) had prior transplantation. The incidence of transplant patients increased temporally (2010: 0.23 %, 2020: 0.36 %, p < 0.001) with *Kidney/Pancreas* comprising the largest proportion (63.5 %). *Non-transplant* more frequently underwent appendectomy and cholecystectomy while transplant patients more commonly received bowel resections. Following entropy balancing*, Liver* was associated with decreased odds of mortality (AOR: 0.67, 95 % CI: 0.54–0.83, Reference: *Non-transplant*). Incremental hospitalization duration was longer in *Liver* and *Heart/Lung* compared to *Non-transplant*. Odds of acute kidney injury, readmissions and costs were higher in all transplant types.

**Conclusion:**

The incidence of transplant recipients undergoing EGS operations has increased. *Liver* was observed to have lower mortality compared to *Non-transplant*. Transplant recipient status, regardless of organ, was associated with greater resource utilization and non-elective readmissions. Multidisciplinary care coordination is warranted to mitigate outcomes in this high-risk population.

## Introduction

Transplantation remains the gold standard surgical treatment for end-stage dysfunction of several organs. Accordingly, the number of transplants performed annually continues to rise with a >3-fold increase from 1988 to 2022 [1]. Advancements in surgical technique, perioperative management and immunosuppression strategies have yielded significantly improved survival following transplantation [2]. Consequently, transplant recipients are now more commonly encountered in clinical practice. Importantly, they may ultimately require care for non-transplant related issues such as emergency general surgery (EGS) conditions, which include benign biliary disease, conditions involving lysis of intra-abdominal adhesions or urgent bowel resection, appendicitis and perforated ulcers [3,4].

Interestingly, transplant recipients have recently been shown to more commonly suffer from EGS conditions, demonstrating a 3.6-fold higher incidence compared to age-matched counterparts [5]. The need for lifelong immunosuppression and greater burden of comorbidities have been cited as potential reasons for this observation among transplant recipients. A systematic review by de'Angelis et al. revealed that perioperative morbidity was high in this cohort, varying from 13.6 % for biliary diseases to 32.7 % for complicated diverticulitis [6]. Notably, mortality in this group was 5.5 % with overwhelming sepsis cited as the most common cause.

Although prior studies have sought to characterize the volume and outcomes of transplant recipients requiring EGS operations, they are largely limited to single centers or evaluate single conditions [5,[7], [8], [9]]. Additionally, data regarding the cost burden of hospitalizations and readmissions, is lacking. The present population-based study sought to evaluate the association of transplant recipient history on clinical and financial outcomes following EGS operations. We hypothesized that prior transplantation would be associated with significantly higher in-hospital mortality, resource utilization and readmissions.

## Methods

The present study was a retrospective analysis of the 2010–2020 Nationwide Readmissions Database (NRD). The NRD is an all-payer, nationally representative inpatient database maintained by the Agency for Healthcare Research and Quality (AHRQ) as part of the Healthcare Cost and Utilization Project (HCUP). The NRD provides estimates for approximately 59 % of all inpatient hospitalizations in the United States annually [10]. The database contains linkage numbers for all sampled patients, allowing for readmissions within each calendar year to be tracked across participating hospitals. The study was deemed exempt from full review by the Institutional Review Board at the University of California, Los Angeles due to its deidentified nature.

Using relevant *International Classification of Disease, Ninth and Tenth Revisions* (ICD-9/10) codes, hospitalizations for adults (≥18 years) who underwent common emergency general surgery operations were identified. Procedures of interest included large/small bowel resection, repair of perforated ulcer, cholecystectomy, appendectomy and lysis of adhesions, as previously reported [3,4,11]. Patients with a history of transplant were stratified as *Kidney/Pancreas*, *Liver* and *Heart/Lung* [12]. Pancreas was grouped with kidney transplants due to the high rates of concomitant kidney-pancreas transplantation [13]. Patients who were admitted electively or were liver transplant recipients who underwent cholecystectomy were excluded (35.5 % omitted).

Patient and hospital characteristics such as age, sex, payer type, income level and teaching status were defined according to the NRD data dictionary. Comorbidities were tabulated using relevant ICD-9/10 diagnosis codes [14]. The modified Elixhauser Comorbidity Index, a previously validated composite score of 30 comorbidities using ICD coding, was used to quantify the burden of chronic conditions with higher scores corresponding to greater severity [15]. To account for center experience, hospitals were divided into tertiles based on institutional EGS operation caseload (low, medium, high volume). Diagnosis-Related Groups in combination with ICD codes were used to identify principal readmission diagnoses [16]. The primary outcome was in-hospital mortality. Secondary outcomes included perioperative complications, intensive care unit (ICU) admission, hospitalization costs, length of stay, and 30-day non-elective readmission. We also sought to evaluate factors associated with transplant-related complications (rejection, failure, graft infection). Complications of interest included stroke, cardiac (arrest, ventricular arrhythmia, tamponade), thrombotic (deep vein thrombosis, pulmonary embolism), respiratory (pneumonia, acute respiratory failure, prolonged mechanical ventilation >96 h), infectious (sepsis, septicemia, surgical site infection), and acute kidney injury [17]. Costs were calculated by application of hospital-specific cost-to-charge ratios to overall hospitalization charges provided by HCUP with inflation adjustment using the 2020 Bureau of Labor Statistics Consumer Price Index [18].

All statistical analyses were performed using Stata 16.1 (StataCorp, College Station, TX) using survey-specific methods to account for clustering. Significance of temporal trends was analyzed using a non-parametric test developed by Cuzick (NP-trend) [19]. Continuous variables are reported as median with interquartile range (IQR) while categorical variables are reported as proportions. Cohort characteristics were compared among cohorts using the Pearson chi-square and Mann-Whitney-*U* tests, where appropriate. Multivariable logistic and linear regression models were developed to evaluate the association of transplant type with outcomes of interest. To account for inter-group differences, entropy balancing was employed to obtain a weighted comparison group with similar covariate distributions. This methodology does not rely on matching propensity scores and therefore maintains the complete cohort, as described elsewhere [[20], [21], [22]]. Standard mean differences (SMDs) were obtained to demonstrate effect size with SMD >0.1 considered significant. Elastic Net with retention of clinically relevant characteristics was used for variable selection [23]. Briefly, Elastic Net regression is a type of machine learning approach that reduces collinearity and selection bias while enhancing generalizability of models. Regression outcomes are reported as adjusted odds ratios (AOR) and β-coefficient (β) for dichotomous and continuous variables, respectively. Statistical significance was defined as α < 0.05.

## Results

**Fig. 1:**
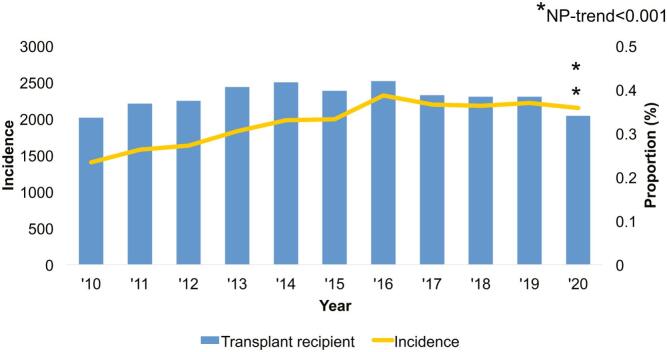
Incidence of prior transplant recipients undergoing emergency general surgery operations over the study period.

**Fig. 2:**
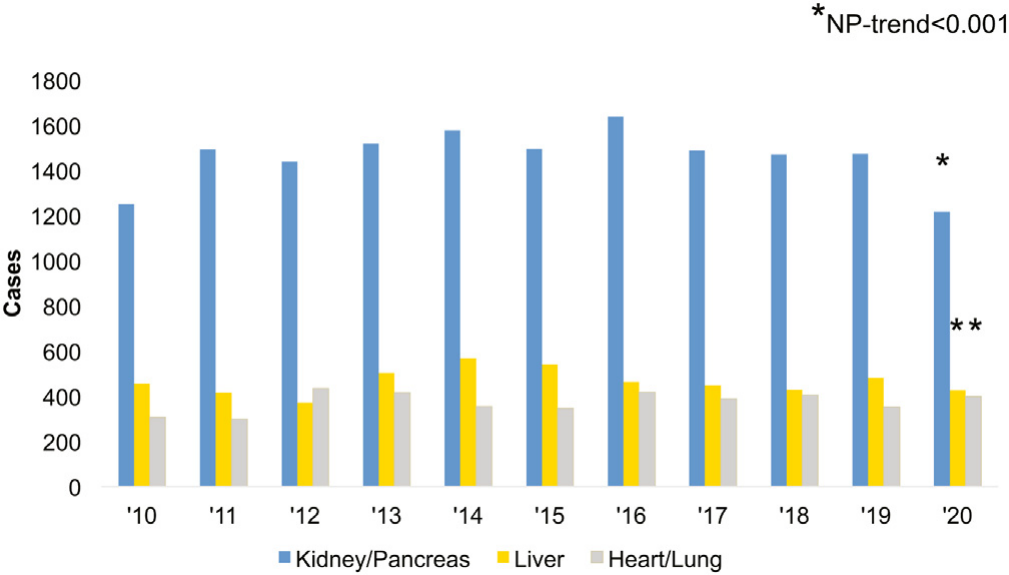
Distribution of transplant type over the study period.

Of an estimated 7,914,815 patients included for analysis, 25,278 (0.32 %) had a history of prior transplant. Over the study period, there was an increased incidence of total transplant recipients (2010–0.23 %, 2020–0.36 %, NP-trend<0.001) with *Kidney/Pancreas* comprising the largest proportion (63.5 %) (Fig. 1). The proportion of *Kidney/Pancreas* decreased significantly over time (2010–62.1 %, 2020–59.7 %, NP-trend<0.001) while *Heart/Lung* increased over the same time period (2010–15.2 %, 2020–19.5 %, NP-trend<0.001) (Fig. 2).

As shown in Table 1, transplant patients were more commonly older, male sex, in the highest income quartile, and insured by Medicare compared to *Non-transplant*. They had a greater burden of comorbidities measured by the Elixhauser Index as well as a greater prevalence of a number of comorbidities including congestive heart failure, hypertension, diabetes, and chronic kidney disease. In addition, they were more commonly managed at large, urban teaching hospitals and those who comprised the highest tertile of EGS volume. Cholecystectomy was the most commonly performed operation in all groups except for *Liver* while small bowel resection and perforated ulcer repair tended to be performed less commonly. *Non-transplant* more frequently underwent appendectomy and cholecystectomy while transplant patients more commonly received bowel resections. While the median day to operation did not differ clinically among cohorts, transplant patients (*Kidney/Pancreas*: 1 (IQR:0–4), *Liver*: 1 (0–4), *Heart/Lung*: (0–4) vs *Non-transplant*: 1 (0–2)) tended to have more patients operated on in later days (p < 0.001).

**Table 1:** Demographic comparison of patient cohort comparing differences among *Non-transplant*, *Kidney/Pancreas*, *Liver* and *Heart/Lung*.

	*Non-transplant* (n = 7,889,537)	*Kidney/Pancreas* (n = 16,058)	*Liver* (n = 5106)	*Heart/Lung* (n = 4114)	p-Value
Age	56 (39–71)	58 (47–67)	61 (53–68)	62 (52–69)	<0.001
Elixhauser Comorbidity Index	2 (1–4)	4 (3–5)	5 (3–6)	4 (2–5)	<0.001
Female sex	57.0	44.3	40.5	32.0	<0.001
Primary payer					<0.001
Private	35.3	25.4	31.3	27.1	
Medicare	38.5	66.6	56.1	64.4	
Medicaid	14.9	5.87	9.59	5.41	
Other payer	11.2	2.08	2.97	3.05	
Income quartile (percentile)					<0.001
76th–100th	20.7	22.0	22.6	23.5	
51st-75th	24.7	25.4	27.3	26.7	
26th–50th	26.3	25.6	26.5	27.2	
0th–25th	28.3	26.9	23.6	22.5	
Hospital bed size					<0.001
Large	58.8	75.7	77.1	86.1	
Medium	26.6	17.6	17.0	10.0	
Small	14.6	6.71	5.92	3.89	
Hospital teaching status					<0.001
Non-metropolitan	9.37	3.50	2.23	2.28	
Metropolitan non-teaching	35.1	20.2	12.2	13.5	
Metropolitan teaching	55.5	76.3	85.5	84.2	
Hospital volume					<0.001
Low	4.54	1.28	1.29	1.82	
Medium	24.5	13.2	10.2	8.47	
High	70.9	85.6	88.6	89.7	
Medical conditions					
Congestive heart failure	8.13	14.7	8.52	20.4	<0.001
Coronary artery disease	12.4	22.5	13.6	21.8	<0.001
Arrhythmia	16.1	22.0	21.2	24.8	<0.001
Valve disorder	3.68	6.53	4.13	4.14	<0.001
Pulmonary hypertension	2.18	3.80	3.62	5.77	<0.001
Peripheral vascular disease	5.64	9.45	8.98	11.0	<0.001
Hypertension	30.2	64.3	45.3	57.4	<0.001
Chronic lung disorder	15.4	11.1	12.3	18.9	<0.001
Diabetes	17.9	43.0	32.2	37.1	<0.001
Hypothyroidism	9.76	12.0	13.4	14.6	<0.001
Liver disease	7.09	6.88	100.0	6.70	<0.001
Coagulopathy	5.23	10.4	17.6	10.1	<0.001
Weight loss	9.04	14.5	22.3	16.2	<0.001
Late kidney disease	1.78	96.6	16.2	13.2	<0.001
Electrolyte imbalance	29.7	47.2	44.7	41.2	<0.001
Anemia	4.74	5.27	7.12	6.17	<0.001
Psychiatric disorders	10.6	10.1	15.9	15.2	<0.001
Operation type					<0.001
Large bowel resection	14.4	17.4	18.1	20.0	
Small bowel resection	7.36	12.3	19.9	10.4	
Peptic ulcer repair	7.48	9.01	15.6	7.95	
Lysis of adhesions	10.3	17.8	29.1	9.53	
Cholecystectomy	40.9	32.4	0.00	40.1	
Appendectomy	19.5	11.1	17.3	12.1	

On unadjusted analysis, rates of in-hospital mortality were greater in prior transplant recipients compared to non-transplant, regardless of graft type (Table 2). Large and small bowel resections had greater rates of mortality as demonstrated in Supplemental Table 1. As shown in Table 2, a number of in-hospital complications and intensive care admission tended to be more common in transplant patients. *Kidney/Pancreas* had the highest rate of transplant-related complications compared to other groups (*Kidney/Pancreas*: 5.91 %, *Liver*: 3.10 %, *Heart/Lung*: 3.53 %, p < 0.001). Importantly, index duration of hospitalization and costs were significantly greater among transplant patients. Rates of non-elective readmission within 30 days were greater in transplant patients with gastrointestinal etiologies comprising the most common primary cause of readmission (Table 3). Notably, renal/genitourinary and endocrine causes tended to be greater reasons for readmission for transplant recipients.

**Table 2:** Unadjusted outcomes comparing *Non-transplant*, *Kidney/Pancreas*, *Liver* and *Heart/Lung*. ICU: intensive care unit.

	*Non-transplant* (n = 7,889,537)	*Kidney/Pancreas* (n = 16,058)	*Liver* (n = 5106)	*Heart/Lung* (n = 4114)	p-Value
Mortality	2.79	4.62	4.36	4.21	<0.001
Stroke	0.47	0.75	0.59	0.33	0.026
Cardiac complications	1.57	3.00	1.55	2.31	<0.001
Thrombotic complications	1.05	1.56	1.47	1.50	<0.001
Respiratory complications	9.58	14.0	14.8	14.6	<0.001
Infectious complications	12.0	20.8	20.4	17.7	<0.001
Acute kidney injury	12.4	23.0	32.5	34.5	<0.001
Need for blood transfusion	11.5	18.0	24.3	17.0	<0.001
Intensive care unit admission	8.08	14.0	17.6	12.5	<0.001
Costs ($1000)	14.5 (9.6–24.6)	22.9 (13.9–40.2)	27.4 (15.2–52.4)	23.7 (14.5–43.9)	<0.001
Length of stay	4 (2–8)	7 (4–13)	8 (4–16)	7 (4–13)	<0.001
30-day non-elective readmission	9.18	19.3	21.0	16.7	<0.001

**Table 3:** Causes of nonelective readmission within 30 days between treatment groups.

	*Non-transplant* (n = 703,936)	*Kidney/Pancreas* (n = 2953)	*Liver* (n = 1026)	*Heart/Lung* (n = 658)	p-Value
Neurologic	1.66	1.86	1.06	0.98	0.60
Psychiatric	1.09	0.16	0.33	1.08	0.007
Cardiovascular	10.2	9.28	8.78	9.40	0.63
Pulmonary	5.50	3.34	4.59	13.7	<0.001
Gastrointestinal	37.9	29.5	31.9	34.4	<0.001
Renal/genitourinary	2.77	3.85	6.38	5.34	<0.001
Infectious	21.2	23.8	23.2	16.5	0.03
Hematologic	1.46	2.47	2.89	0.40	0.001
Endocrine	2.99	4.02	5.11	3.62	0.021
Musculoskeletal	1.23	1.86	1.39	1.94	0.25

On adjusted analysis, *Liver* had a lower odds of transplant-related complications (AOR: 0.53, 95 % CI: 0.37–0.76) while *Heart/Lung* had greater odds (AOR: 1.30, 95 % CI: 1.05–0.1.61) with reference to *Kidney/Pancreas* (Table 4). Others factors associated with transplant-related complications included undergoing lysis of adhesions (AOR: 1.55, 95 % CI: 1.17–2.05, Reference: large bowel resection), hypertension and coagulopathy. Female sex, higher EGS volume tertiles (High: AOR: 0.42, 95 % CI: 0.19–0.93; Medium: AOR: 0.45, 95 % CI: 0.21–0.98, Reference: Low) and urban non-teaching status (Reference: urban teaching) were factors associated with lower odds of transplant-related complications.

**Table 4:** Factors associated with transplant-related complications following multivariable adjustment.

	Adjusted odds ratio	95 % CI	p-Value
Transplant type			
*Kidney/Pancreas*	1 (ref.)		
*Liver*	0.53	0.37–0.76	0.001
*Heart/Lung*	1.30	1.05–1.61	0.017
Age per year	0.99	0.98–0.99	0.001
Female sex	0.79	0.65–0.95	0.012
Elixhauser comorbidity index	0.97	0.90–1.05	0.45
Income quartile			
76th–100th	1 (ref.)		
51st-75th	1.22	0.86–1.46	0.39
26th–50th	0.96	0.74–1.27	0.79
0th–25th	0.91	0.69–1.19	0.49
Payer status			
Private	1 (ref.)		
Medicare	1.17	0.93–1.49	0.18
Medicaid	1.16	0.77–1.75	0.47
Other	0.68	0.29–1.56	0.36
Hospital bed size			
Large	1 (ref.)		
Medium	0.76	0.56–1.03	0.079
Small	0.68	0.44–1.05	0.079
Hospital teaching status			
Metropolitan teaching	1 (ref.)		
Metropolitan non-teaching	0.54	0.40–0.72	<0.001
Non-metropolitan	0.35	0.12–1.04	0.058
Hospital volume			
Low	1 (ref.)		
Medium	0.45	0.21–0.98	0.043
High	0.42	0.20–0.93	0.031
Operation type			
Large bowel resection	1 (ref.)		
Small bowel resection	1.11	0.84–1.48	0.47
Peptic ulcer repair	0.70	0.52–0.93	0.014
Lysis of adhesions	1.55	1.17–2.05	0.003
Cholecystectomy	0.70	0.52–0.93	0.014
Appendectomy	0.68	0.44–1.05	0.081
Medical conditions			
Congestive heart failure	1.21	0.94–1.56	0.13
Coronary artery disease	1.09	0.87–1.36	0.44
Arrhythmia	1.13	0.88–1.45	0.34
Valve disorder	1.03	0.60–1.79	0.91
Hypertension	1.50	1.19–1.88	0.001
Chronic lung disorder	0.74	0.53–1.01	0.06
Diabetes	1.22	0.98–1.51	0.07
Coagulopathy	1.66	1.26–2.19	<0.001
Weight loss	1.45	1.15–1.82	0.002
Electrolyte imbalance	1.67	1.34–2.08	<0.001

**Fig. 3:**
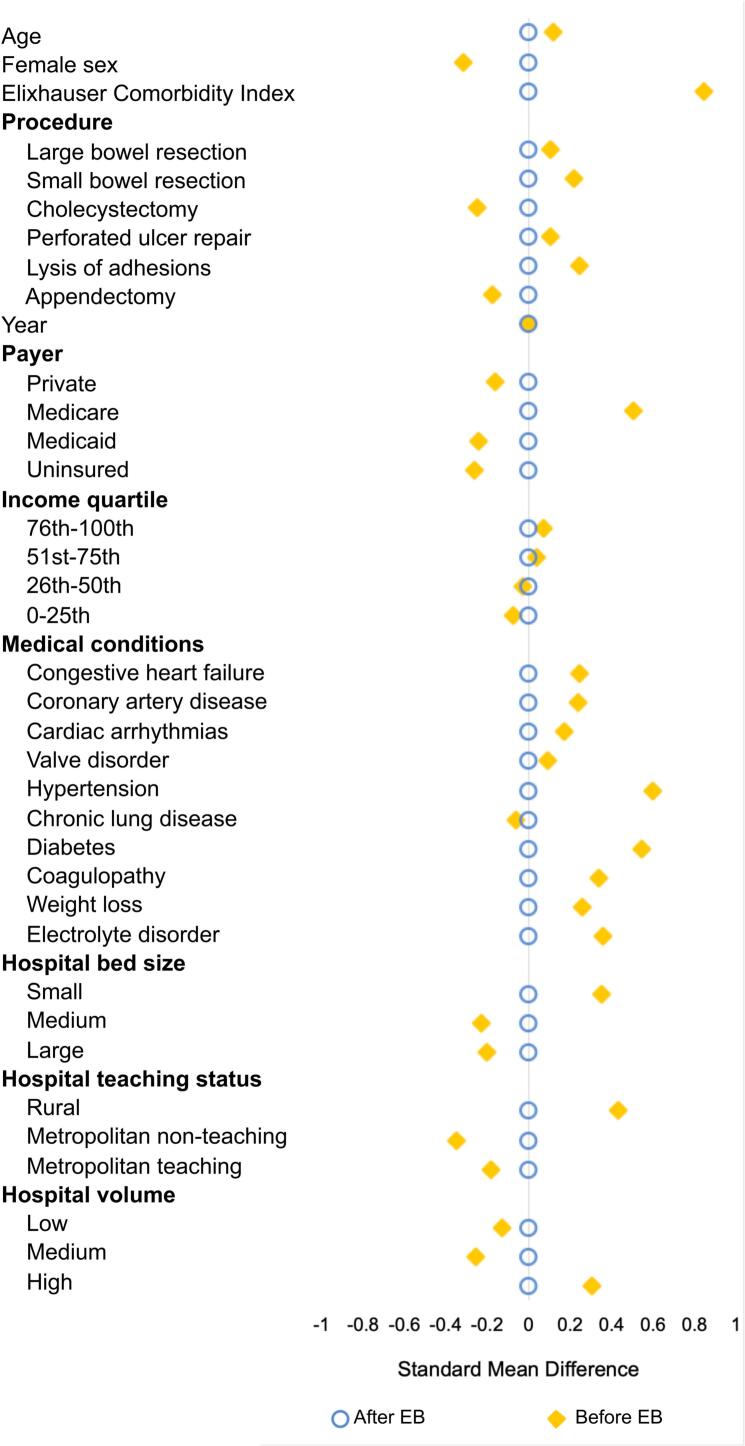
Pre- and post- covariate balancing after entropy balancing. EB: entropy balancing.

Following adjustment with entropy balancing and achieving acceptable covariate balance (Fig. 3), *Liver* was associated with decreased odds of mortality (AOR: 0.67, 95 % CI: 0.54–0.83) while *Kidney/Pancreas* and *Heart/Lung* demonstrated similar odds compared to *Non-transplant* counterparts (Table 5). Interestingly, *Liver* was also associated with lower likelihood of cardiac, thrombotic and respiratory complications. *Kidney/Pancreas* was associated with greater odds of infectious complications compared to *Non-transplant*. While *Kidney/Pancreas* had similar incremental hospitalization duration compared to *Non-transplant*, *Liver* and *Heart/Lung* had longer length of stay. Acute kidney injury, costs and odds of 30-day non-elective readmissions were higher in transplant patients, regardless of graft type. Sensitivity analysis was performed without entropy balancing. As shown in Supplemental Table 2, adjusted outcomes were maintained. Interestingly, *Kidney* became associated with greater odds of in-hospital mortality (AOR: 1.26, 95 % CI: 1.10–1.44), cardiac complications, and longer incremental length of stay (β: 0.8 days, 95 % CI: 0.5–1.1) with reference to *Non-transplant*.

**Table 5:** Adjusted outcomes among treatment groups with *Non-transplant* as reference. ICU: intensive care unit.

	AOR/β^a^	95 % CI			p-Value
Mortality					
*Kidney/Pancreas*	1.11	0.97	–	1.26	0.12
*Liver*	0.67	0.54	–	0.83	<0.001
*Heart/Lung*	0.99	0.77	–	1.29	0.97
Stroke					
*Kidney/Pancreas*	1.00	0.72	–	1.40	0.99
*Liver*	0.50	0.21	–	1.16	0.11
*Heart/Lung*	0.42	0.19	–	0.90	0.026
Cardiac complications					
*Kidney/Pancreas*	1.10	0.93	–	1.30	0.25
*Liver*	0.50	0.35	–	0.72	<0.001
*Heart/Lung*	0.84	0.61	–	1.16	0.29
Thrombotic complications					
*Kidney/Pancreas*	0.83	0.67	–	1.02	0.07
*Liver*	0.45	0.29	–	0.69	<0.001
*Heart/Lung*	0.83	0.56	–	1.24	0.37
Respiratory complications					
*Kidney/Pancreas*	0.96	0.88	–	1.05	0.38
*Liver*	0.78	0.68	–	0.91	0.001
*Heart/Lung*	1.04	0.88	–	1.23	0.63
Infectious complications					
*Kidney/Pancreas*	1.21	1.13	–	1.30	<0.001
*Liver*	0.95	0.83	–	1.08	0.40
*Heart/Lung*	0.95	0.82	–	1.10	0.49
Acute kidney injury					
*Kidney/Pancreas*	1.11	1.04	–	1.19	0.002
*Liver*	1.69	1.51	–	1.89	<0.001
*Heart/Lung*	2.20	1.95	–	2.47	<0.001
Need for blood transfusion					
*Kidney/Pancreas*	0.99	0.92	–	1.08	0.96
*Liver*	1.04	0.92	–	1.19	0.52
*Heart/Lung*	1.13	0.96	–	1.32	0.13
Intensive care unit admission					
*Kidney/Pancreas*	0.99	0.92	–	1.09	0.95
*Liver*	0.96	0.83	–	1.10	0.55
*Heart/Lung*	0.94	0.79	–	1.12	0.51
Adjusted costs ($1000)					
*Kidney/Pancreas*	2.2	0.7	–	3.7	0.005
*Liver*	14.9	9.5	–	20.3	<0.001
*Heart/Lung*	6.8	3.8	–	9.8	<0.001
Length of stay (days)					
*Kidney/Pancreas*	0.3	−0.03	–	0.6	0.08
*Liver*	1.9	1.0	–	2.8	<0.001
*Heart/Lung*	1.0	0.3	–	1.7	<0.001
30-day non-elective readmission					
*Kidney/Pancreas*	1.33	1.24	–	1.42	<0.001
*Liver*	1.39	1.22	–	1.58	<0.001
*Heart/Lung*	1.22	1.06	–	1.41	0.005

a Binary outcomes reported as adjusted odds ratio (AOR) while continuous outcomes reported as β coefficient (β).

Subsequent sensitivity analysis was performed with all patients with solid transplant history was binned into one group. As shown in Supplemental Table 3, the observed findings were similar. Compared to *Non-transplant*, *Transplant* status was not associated with in-hospital mortality. While the odds of thrombotic and respiratory complications were lower in *Transplant*, the odds of infectious complications and acute kidney injury were higher in transplant patients with *Non-transplant* as reference. Importantly, resource utilization and odds of 30-day non-elective readmission were persistently higher in *Transplant* compared to *Non-transplant*.

## Discussion

With significant improvements in perioperative care of transplant recipients, more are surviving their respective end-stage organ diseases to ultimately require emergency general surgery operations. The present study used a large, nationally-representative cohort to evaluate the association of transplant status on clinical outcomes and had many notable findings. Prior liver transplant recipients appeared to have lower mortality and complication rates compared to non-transplant patients. Importantly, resource utilization was generally greater for prior transplant recipients. Finally, the transplant cohort demonstrated significantly greater odds of non-elective readmissions. Our findings warrant further discussion.

In contrast to a study by Bhatti et al. which found greater mortality in transplant recipients following EGS, the present work found that transplant status was not significantly associated with increased odds of mortality [24]. Interestingly, liver transplant recipients were observed to have lower odds of mortality and a number of other complications, including transplant-related complications, after adjustment. There are a multitude of factors that may explain this discrepancy. Importantly, Bhatti and others evaluated outcomes for transplant patients in the immediate peritransplant period. Given the requirement for high-dose immunosuppression, it may directly contribute to a more complex clinical course [5]. Although we do not know the timing of transplant in our cohort, there may be a number of patients who had remote histories of transplant with stable, long-standing immunosuppression. Importantly, it is notable to consider that the proportion of EGS conditions varied between groups. Large and small bowel resections, which are associated with greater mortality than other EGS operations, had greater incidence in non-transplant patients. Therefore, it is possible the type of operation performed may have been a more substantial contributor to mortality than the presence of transplant. Finally, although transplant patients had a greater overall burden of comorbidities, liver transplant patients may have had a less severe disease compared to others. As end stage liver disease does not tend to have as many associated comorbidities and grafts are not as immunogenic as other solid organs, it is possible that these findings may explain disparate outcomes [25]. Importantly, our database does not have sufficient granularity to assess the initial indication of transplant. Based upon the retrospective study design, we can only make associations and can only postulate explanations for our observations. Regardless, future work is needed to better define lower mortality and other complication rates in this cohort.

The present study found that costs were significantly higher in transplant patients, regardless of graft type. These may be caused by a number of factors. The management of perioperative complications may be more complex, requiring greater resource allocation [26]. Although we do not know the role of the transplant team in the management of these individuals, they may have required greater coordination of care regarding medical optimization of comorbidities and immunosuppression regimens. Because transplant patients tended to undergo their operations in later hospitalization days compared to non-transplant, it is possible they required additional work-up prior to surgical treatment. A prior study from our group demonstrated greater costs in kidney transplant recipients following cardiac operations and transplant recipients following hip arthroplasty [12,27]. Taken together, our findings highlight the resource burden in this population. Efforts must be performed to limit excessive costs in this population while still providing cost-conscious care.

We observed high overall rates of non-elective readmissions in transplant recipients and had significantly higher odds of readmission compared to non-transplant patients. Additionally, we found that renal/genitourinary and gastrointestinal causes comprised the most significant proportion of primary reason for rehospitalization. Given the higher odds of acute kidney injury in the index hospitalization, requiring further hospitalization for continued care of renal dysfunction is possible. In addition, agents such as tacrolimus, which comprise the most common long-term immunosuppressant in this population, may contribute to further nephrotoxicity in the perioperative period [28]. Although beyond the scope of the study, care fragmentation, defined as requiring readmission at a hospital different than the index procedure, remains particularly salient in this population. Given the need for perioperative optimization and need for continued immunosuppression management, transplant patients may be particularly harmed by care fragmentation. It has previously been shown to adversely affect short-term survival following liver transplantation [29]. Further studies evaluating the impact of readmissions on long-term outcomes in this population are needed.

The present study has several limitations inherent to its nature as an administrative database. Transplant status was reliant on ICD-9/10 codes which does not specify the time in which a patient received the transplant. Additionally, there is a lack of granularity regarding the patient's clinical course prior to operation. We do not know the timing of the diagnosis and therefore do not know if a perioperative complication occurred prior to their procedure. Importantly, patients managed solely non-operatively were not assessed. Furthermore, there is no information regarding what immunosuppression regimen patients were prescribed or indication of the initial transplant which may affect their risk of developing perioperative complications. Finally, we are unable to determine the presence of a transplant team in the management or if patients were managed at the same hospital in which they received the transplant. Despite these limitations, we present the largest modern cohort of transplant patients undergoing EGS operations and used validated methodologies to report outcomes.

In conclusion, the incidence of transplant recipients undergoing emergency general surgery operations has risen. Liver transplant patients were observed to have lower mortality and other complications, including transplant-related rejection, graft failure and infection compared to others. Transplant recipient status, regardless of organ, was demonstrated to be associated with greater odds of acute kidney injury, resource utilization and non-elective readmissions. When managing this unique patient population, special consideration must be applied to limit morbidity and resource burden.

## Funding/financial support

No sources of funding applicable for this work.

## Ethical approval statement

Exempt from full review by the Institutional Review Board at the University of California, Los Angeles due to the deidentified nature of the dataset

## CRediT authorship contribution statement

Study conception and design: Z.T., S.S.B.
Acquisition of data: Z.T., S.R., P.B.
Analysis and interpretation of data: Z.T., J.L, S.R., S.S.B., L.S., P.B.
Drafting of manuscript: Z.T., J.L, L.S., P.B.
Critical revision: all authors.
Supervision: P.B.

## Declaration of competing interest

P.B. is a surgical proctor for Atricure Inc. (Irvine, CA) and received consulting fees unrelated to the present work. All other authors have no disclosures.

## Appendix A Supplementary data

Supplementary tables Image 1
