# Supplementary material for: Clinical and financial outcomes of transplant recipients following emergency general surgery operations
Source: Surg Open Sci. 2023 Apr 15;13:41–7. doi: 10.1016/j.sopen.2023.04.002 (PMC10149279; doi:10.1016/j.sopen.2023.04.002)
Supplement: Supplementary file 1 — Supplementary tables [file mmc1.docx]

Supplemental Table 1. Unadjusted mortality rates of EGS operations

|  | Mortality |
| --- | --- |
| Large bowel resection | 8.11 |
| Small bowel resection | 7.74 |
| Peptic ulcer repair | 5.93 |
| Lysis of adhesions | 2.93 |
| Cholecystectomy | 0.64 |
| Appendectomy | 0.22 |

Supplemental Table 2. Adjusted outcomes among treatment groups with *Non-transplant* as reference without entropy balancing. *Binary outcomes reported as adjusted odds ratio (AOR) while continuous outcomes reported as β coefficient (β).

|  | AOR/ β* | 95% CI | | | *p-value* |
| --- | --- | --- | --- | --- | --- |
| Mortality |  |  |  |  |  |
| *Kidney/Pancreas* | 1.26 | 1.10 | - | 1.44 | 0.001 |
| *Liver* | 0.69 | 0.56 | - | 0.86 | 0.001 |
| *Heart/Lung* | 1.09 | 0.84 | - | 1.42 | 0.52 |
| Stroke |  |  |  |  |  |
| *Kidney/Pancreas* | 1.10 | 0.78 | - | 1.55 | 0.58 |
| *Liver* | 0.52 | 0.23 | - | 1.19 | 0.12 |
| *Heart/Lung* | 0.44 | 0.20 | - | 0.94 | 0.03 |
| Cardiac complications |  |  |  |  |  |
| *Kidney/Pancreas* | 1.19 | 1.00 | - | 1.40 | 0.04 |
| *Liver* | 0.49 | 0.34 | - | 0.70 | <0.001 |
| *Heart/Lung* | 0.91 | 0.65 | - | 1.26 | 0.56 |
| Thrombotic complications |  |  |  |  |  |
| *Kidney/Pancreas* | 0.84 | 0.68 | - | 1.04 | 0.10 |
| *Liver* | 0.41 | 0.26 | - | 0.63 | <0.001 |
| *Heart/Lung* | 0.86 | 0.58 | - | 1.27 | 0.46 |
| Respiratory complications |  |  |  |  |  |
| *Kidney/Pancreas* | 1.05 | 0.96 | - | 1.15 | 0.29 |
| *Liver* | 0.79 | 0.68 | - | 0.92 | 0.002 |
| *Heart/Lung* | 1.07 | 0.90 | - | 1.27 | 0.45 |
| Infectious complications |  |  |  |  |  |
| *Kidney/Pancreas* | 1.27 | 1.17 | - | 1.37 | <0.001 |
| *Liver* | 0.93 | 0.82 | - | 1.06 | 0.3 |
| *Heart/Lung* | 0.96 | 0.84 | - | 1.11 | 0.61 |
| Acute kidney injury |  |  |  |  |  |
| *Kidney/Pancreas* | 1.18 | 1.09 | - | 1.27 | <0.001 |
| *Liver* | 1.68 | 1.48 | - | 1.90 | <0.001 |
| *Heart/Lung* | 2.55 | 2.25 | - | 2.88 | <0.001 |
| Need for blood transfusion |  |  |  |  |  |
| *Kidney/Pancreas* | 1.09 | 0.99 | - | 1.19 | 0.07 |
| *Liver* | 1.03 | 0.90 | - | 1.19 | 0.63 |
| *Heart/Lung* | 1.20 | 0.99 | - | 1.45 | 0.055 |
| Intensive care unit admission |  |  |  |  |  |
| *Kidney/Pancreas* | 1.08 | 0.99 | - | 1.18 | 0.08 |
| *Liver* | 0.96 | 0.83 | - | 1.11 | 0.61 |
| *Heart/Lung* | 0.96 | 0.80 | - | 1.16 | 0.70 |
| Adjusted costs ($1000) |  |  |  |  |  |
| *Kidney/Pancreas* | 5.3 | 3.8 | - | 6.7 | <0.001 |
| *Liver* | 20.1 | 13.7 | - | 26.6 | <0.001 |
| *Heart/Lung* | 9.6 | 6.5 | - | 12.6 | <0.001 |
| Length of stay |  |  |  |  |  |
| *Kidney/Pancreas* | 0.8 | 0.5 | - | 1.1 | <0.001 |
| *Liver* | 2.6 | 1.6 | - | 3.6 | <0.001 |
| *Heart/Lung* | 1.4 | 0.7 | - | 2.1 | <0.001 |
| 30-day non-elective readmission |  |  |  |  |  |
| *Kidney/Pancreas* | 1.48 | 1.38 | - | 1.59 | <0.001 |
| *Liver* | 1.41 | 1.24 | - | 1.59 | <0.001 |
| *Heart/Lung* | 1.37 | 1.18 | - | 1.59 | <0.001 |

|  | Adjusted Odds Ratio / β Coefficient* | | | | |
| --- | --- | --- | --- | --- | --- |
|  | *Transplant* | 95% CI | | | *p-value* |
| Mortality | 0.97 | 0.87 | - | 1.07 | 0.53 |
| Stroke | 0.78 | 0.58 | - | 1.05 | 0.098 |
| Cardiac complications | 0.92 | 0.81 | - | 1.06 | 0.26 |
| Thrombotic complications | 0.71 | 0.60 | - | 0.84 | <0.001 |
| Respiratory complications | 0.93 | 0.87 | - | 0.99 | 0.04 |
| Infectious complications | 1.11 | 1.04 | - | 1.17 | 0.001 |
| Acute kidney injury | 1.37 | 1.30 | - | 1.44 | <0.001 |
| Need for blood transfusion | 1.03 | 0.97 | - | 1.09 | 0.40 |
| Intensive care unit admission | 0.98 | 0.92 | - | 1.05 | 0.55 |
| Adjusted costs ($1000) | 5.5 | 4.0 | - | 7.0 | <0.001 |
| Length of stay | 0.7 | 0.4 | - | 1.0 | <0.001 |
| 30-day non-elective readmission | 1.32 | 1.25 | - | 1.4 | <0.001 |

Supplemental Table 3. Sensitivity analysis of adjusted outcomes with patients with history of transplant binned into one category with *Non-transplant* as reference. *Binary outcomes reported as adjusted odds ratio (AOR) while continuous outcomes reported as β coefficient (β).
